# Koopmans' Analysis of Chemical Hardness with Spectral-Like Resolution

**DOI:** 10.1155/2013/348415

**Published:** 2013-07-18

**Authors:** Mihai V. Putz

**Affiliations:** Laboratory of Structural and Computational Chemistry, Biology-Chemistry Department, West University of Timişoara, Pestalozzi Street No. 16, 300115 Timişoara, Romania

## Abstract

Three approximation levels of Koopmans' theorem are explored and applied: the first referring to the inner quantum behavior of the orbitalic energies that depart from the genuine ones in Fock space when the wave-functions' Hilbert-Banach basis set is specified to solve the many-electronic spectra of spin-orbitals' eigenstates; it is the most subtle issue regarding Koopmans' theorem as it brings many critics and refutation in the last decades, yet it is shown here as an irrefutable “observational” effect through computation, specific to any in silico spectra of an eigenproblem; the second level assumes the “frozen spin-orbitals” approximation during the extracting or adding of electrons to the frontier of the chemical system through the ionization and affinity processes, respectively; this approximation is nevertheless workable for great deal of chemical compounds, especially organic systems, and is justified for chemical reactivity and aromaticity hierarchies in an homologue series; the third and the most severe approximation regards the extension of the second one to superior orders of ionization and affinities, here studied at the level of chemical hardness compact-finite expressions up to spectral-like resolution for a paradigmatic set of aromatic carbohydrates.

## 1. Introduction

In modern structural chemistry [1], the Aufbau principle for atomic periodicity [2], Hückel molecular orbital theory [3], reinforced by Hartree-Fock theory [4, 5] and then completed by the density functional theory (DFT) [6–12], are all cornerstones in modeling and predict physical behavior of many electronic systems, from atoms to molecules and solid states. However, the chemical regime of this wide physical range of many-electronic manifestation was often reduced to the frontier electrons [13, 14] targeting the chemical reactivity as a special and specific way of interaction thus defining a proper chemical orthogonal space, while generalizing the custom Cartesian-physical one [15]. In this context, the density functional theory (DFT) developed in the celebrated conceptual chemical reactivity theory where, for instance, the classical concept of electronegativity [16, 17] acquired new and fruitful shape and formulation [18, 19] along promoting other informational indices as such chemical hardness [20–24], chemical action [25, 26], and electrophilicity [27–29], with the allied principles [30–36] which ultimately delivered the present rationalization of chemical bond by min-max variational principles in a coupled interrelation within the bonding chemical scenario [37, 38]. However, apart from these somehow global frontier indices, there remains the inquiring behavior of frontier orbitals themselves, during the electronic charge transfer encountering in chemical reactivity, which, at the limit, obey the Koopmans theorem (KT) [39], since approximately not depending on the number of electrons they host in the course of chemical reaction [14, 40]. Of course, historically, the Koopmans theorem was developed within Hartree-Fock theory giving the route to predict ionization potentials [41], while giving simple physical interpretation to the eigen value of the Fock matrix and justifying the existence of the “orbitals” by their observable energies [42, 43]. 

Although criticized [44, 45] because its inner definition excludes electronic relaxation effects at the orbitals' levels, that is, neglecting the electronic correlation, Koopmans theorem resisted through time since proving versatile ways for avoiding or including the “missing correlation information” by remarkable methods. Among most preeminent approaches in this direction are

- the electron propagation theory (through considering the self-energy operator) [46];
- disproving the existence of ionization potential as the lowest eigenvalues of KT but generalizing it to the arbitrarily close value to IP [43];
- interpreting the self-consistent Hartree-Fock field as coupled harmonic oscillators evolving in a nonlinear potential [47];
- variationally extending KT to restricted open-sells canonical orbitals which nevertheless overestimate the Aufbau principle [48];
- differentiating between vertical and adiabatic ionization potentials for the strongest line of the band and for the 0 → 0 band transition, respectively [49];
- establishing the connection with DFT through Janak's theorem and proving its reliability for large molecular systems (including fullerenes or boron nitride nanotubes B_48_N_48_) [50];
- including the negative electron affinity extensions within DFT for halogenated small organic molecules [51];
- establishing the direct connection of the frontier orbitals with the pi-electrons and of the electronic transfer of conjugated aromatic systems [52];
- driving the electronic transfer in alfa-substituted organic polymers [53], providing with optical spectra analysis for intervalence complexes formed by organic bridges between radical ions [54];
- till the modeling of anti-inflammatory activities of clinical drugs acting through ionization processes in special [55] and by chemical reactivity indices and DFT in general [56–62].

It is the last context in which the present work is placed too: it reviews the Koopmans method with the Hartree-Fock theory while emphasizing on the generality of the method there where a limitation was previously identified; it will be connected then with chemical hardness by means of LUMO-HOMO gap that eventually cancels the correlations and relaxation opposite effects appareling on the successive highest occupied and lowest unoccupied molecular orbitals; it will end with an illustration on how chemical hardness analysis based on Koopmans superior orders orbitals is fitting with compact-finite difference expression of it in the highest analytical accuracy of spectral-like resolution (SLR) and how these two faces of the chemical hardness generally asses the aromaticity hierarchy along a homological series of organic molecules.

## 2. Reviewing the Koopmans Method

### 2.1. Nature of Koopmans' Theorem within Hartree-Fock Formalism

Consider the many-electronic wave-function ket-vector in *N*-dimensional Hilbert spin-orbitals' space

(1) $$\begin{array}{c} |\Psi_{0}^{\left(N\right)}\rangle =|\psi_{1}\cdots \psi_{a}\cdots \psi_{N}\rangle \end{array}$$

constructed from one-electronic Fock eigenstates

(2) $$\begin{array}{c} \hat{f}|\psi_{a}\rangle =\varepsilon_{a}|\psi_{a}\rangle ,\;a=\bar{1,N} \end{array}$$

with the Fock operator

(3) $$\begin{array}{c} \hat{f}=\hat{h}+\sum_{b=1}^{N}\left[{\hat{J}}_{b}-{\hat{K}}_{b}\right] \end{array}$$

written (in atomic units) in terms of the kinetic + potential operator

(4) $$\begin{array}{c} \hat{h}=-\frac{1}{2}\nabla_{1}^{2}-\sum_{A}\frac{Z_{A}}{r_{1A}} \end{array}$$

accounting for the one-electronic motion in the potential of the nuclei/nucleus in a molecular/atomic system; to it one adds the resulting energy remaining from subtraction of the exchange influence

(5) $$\begin{array}{c} K_{b}\left(1\right)=\int d2\psi_{b}^{*}\left(2\right)r_{12}^{-1}\psi_{b}\left(1\right)=\int d2\psi_{b}^{*}\left(2\right)r_{12}^{-1}{℘}_{12}\psi_{b}\left(2\right) \end{array}$$

from the interelectronic Coulombic interaction

(6) $$\begin{array}{c} J_{b}\left(1\right)=\int d2\psi_{b}^{*}\left(2\right)r_{12}^{-1}\psi_{b}\left(2\right). \end{array}$$

Note in the exchange energy (5) the appearance of the interchanging particle operator *℘*_12_ which acts by interchanging 1↔2 particles on all wave functions on its right-had side; it also helps in identifying the Hartree-Fock potential in the Fock operator rewritten as

(7) f(1)=h(1)+vHF(1)=h(1)+∑b=1N∫d2ψb∗(2)r12−1(1−℘12)ψb(2).

Altogether this formalism allows for expressing the total eigenenergy of the *N*-electronic system to be successively written as follows:

(8) EN=〈Ψ0(N)|H^|Ψ0(N)〉=∑a=1N〈ψa|h^|ψa〉 +12∑a=1,b=1N[〈ψa|∫d2ψb∗(2)r12−1ψb(2)|ψa〉−〈ψa|∫d2ψb∗(2)r12−1℘12ψb(2)|ψa〉]=∑a=1N〈ψa|h^|ψa〉 +12∑a=1,b=1N[∫d1d2ψa∗(1)ψb∗(2)r12−1ψb(2)ψa(1)−∫d1d2ψa∗(1)ψb∗(2)r12−1℘12ψb(2)ψa(1)]=∑a=1N〈ψa|h^|ψa〉 +12∑a=1,b=1N[∫d1d2ψa∗(1)ψb∗(2)r12−1ψb(2)ψa(1)−∫d1d2ψa∗(1)ψb∗(2)r12−1ψb(1)ψa(2)]=∑a=1N〈ψa|h^|ψa〉 +12∑a=1,b=1N[∫d1d2ψa∗(1)ψa(1)r12−1ψb∗(2)ψb(2)−∫d1d2ψa∗(1)ψb(1)r12−1ψb∗(2)ψa(2)]≡∑a=1N〈ψa|h^|ψa〉+12∑a=1,b=1N[〈aa ∣ bb〉−〈ab ∣ ba〉]≡∑a=1N〈a|h^|a〉+12∑a=1,b=1N〈ab ∣ ab〉.

The issue appears while noticing the Fock operator functional dependency on the occupied spin orbitals; once the functions *ψ*_*b*_(2) are known (say as a basis set), $\hat{f}$ becomes a well-defined Hermitic operator with infinite eigenstates and functions: it allows therefore distinction between

- the first lowest *N* spin-orbitals occupied in the overall wave-function |Ψ_0_^(*N*)^〉 = |*ψ*_1_ ⋯ *ψ*_*a*_ ⋯ *ψ*_*N*_〉;
- the rest (from *N* up to infinity) virtual of unoccupied orbitals, formally denoted as *ψ*_*r*_, *ψ*_*s*_,….

In this *computational* context, the orbitals extend their spectrum with the general eigenenergies as follows:

(9) εi=1,…,∞=〈ψi|f^|ψi〉=〈ψi|(h^+∑b=1N[J^b−K^b])|ψi〉=〈ψi|h^|ψi〉+∑b=1N[〈ψi|J^b|ψi〉−〈ψi|K^b|ψi〉]≡〈i|h^|i〉+∑b=1N[〈ii ∣ bb〉−〈ib ∣ bi〉]≡〈i|h^|i〉+∑b=1N〈ib ∣ ib〉.

The important point here is that when turning the last equation into the orbital eigen-energies of the occupied orbitals

(10) $$\begin{array}{c} \varepsilon_{a=1,\ldots ,N}=\langle a\left|\hat{h}\right|a\rangle +\sum_{\begin{array}{c} b=1\\ b\neq a \end{array}}^{N}\langle ab\;|\;ab\rangle \end{array}$$

and of those left unoccupied

(11) $$\begin{array}{c} \varepsilon_{r=N+1,\ldots ,\infty }=\langle r\left|\hat{h}\right|r\rangle +\sum_{b=1}^{N}\langle rb\;|\;rb\rangle , \end{array}$$

the summation upon the energies of the occupied spin orbitals yields the interesting result

(12) $$\begin{array}{c} \sum_{a=1}^{N}\varepsilon_{a}=\sum_{a=1}^{N}\langle a\left|\hat{h}\right|a\rangle +\sum_{a=1,b=1}^{N}\langle ab\;|\;ab\rangle \neq E_{N}. \end{array}$$

Equation (12) does not exactly recovering the previous total energy of the *N*-occupied spin-orbitals Equation (8), when they were considered “free (not depending)” of computation (basis set); however, this may be considered as *in silico* manifestation of quantum “observability” (once a basis set representation applies) which destroys the quantum system in itself's (or eigen) manifestation. Here the mathematical properties of eigenfunction computed upon a given basis on Hilbert-Banach spaces determine the “shift” or the “unrealistic” energies of orbitals since spanning those occupied and unoccupied alike; from the present dichotomy basically follows all critics on the Hartree-Fock formalism and of allied molecular orbital theory, Koopmans' “theorem” included; instead, there seems that such departure of the computed from the exact energy orbitals is inherent to quantum formalism and not necessary a weakness of the Hartree-formalism itself, since it will appear to any quantum many-particle problem involving eigenproblems. 

**Figure 1:**
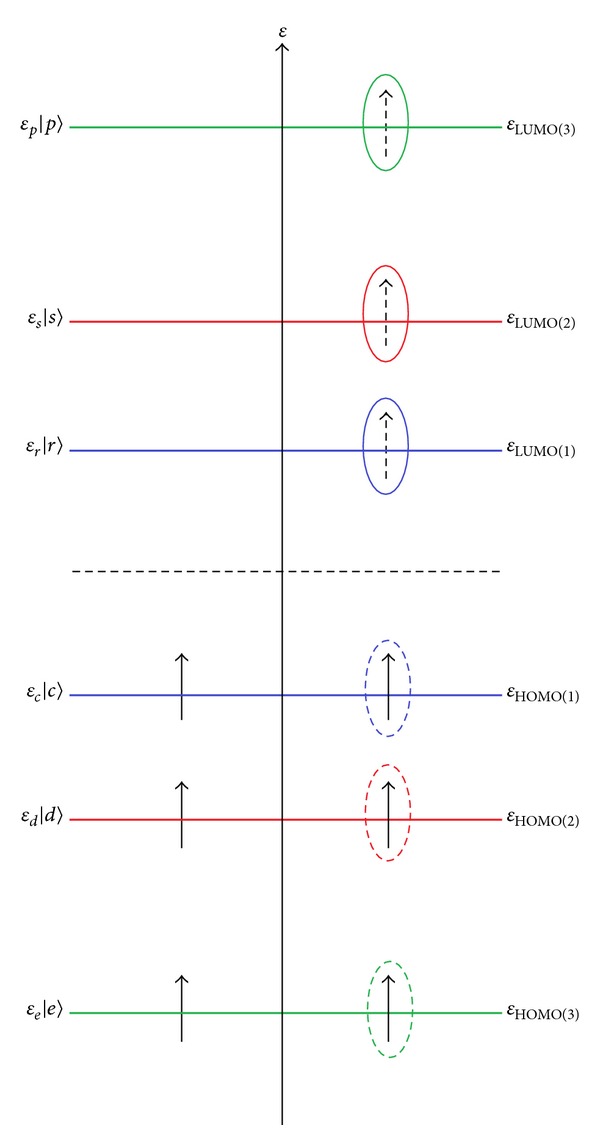
The paradigmatic *in silico* spectra of the first three highest occupied and lowest unoccupied molecular orbitals illustrating the respective, successive, ionization and affinities energies as provided by Koopmans' theorem. Note that KT implies ionization and affinity of one electron on successive levels and not of successive electrons on levels; see the marked occupied and virtual spin orbitals.

Now, returning to the previous occupied and unoccupied orbital energy, one may assume (Koopmans' ansatz) that, on the frontier levels of a many-electronic system, extracting or adding of an electron (or even few of them, but lesser than the total number of valence electrons) will not affect the remaining (or *N* ± 1, *N* ± 1 ± 1′,…, electronic orbitals) states, on successive levels and not successive electrons on levels (see Figure 1). 

This approach allows simplifying of the common terms and emphasizing only on the involving frontier orbitals participating in chemical reactivity. Accordingly, for the first ionization potential one successively obtains (see Figure 1)

(13) IP1=EN−1−EN=〈Ψc(N−1)|H^|Ψc(N−1)〉−〈Ψ0(N)|H^|Ψ0(N)〉={∑a=1a≠cN〈a|h^|a〉+12∑a=1,b=1a≠c,b≠cN〈ab ∣ ab〉} −{[∑a=1a≠cN〈a|h^|a〉+〈c|h^|c〉]   +12[∑a=1,b=1a≠c,b≠cN〈ab ∣ ab〉+∑a=1,b=1a≠c,b=cN〈ac ∣ ac〉      +∑a=1,b=1a=c,b≠cN〈cb ∣ cb〉]}=−〈c|h^|c〉−∑b=1N〈cb ∣ cb〉=−εc=−εHOMO(1).

Remarkable, in this analytics, one starts with *in se* quantum expression of total energies of the *N* and (*N* − 1) systems and ends up with a result characteristic to the computational (shifted) realm since recovering the orbital energy of the *in silico* state from which the electron was removed. Yet, one may ask how such *in se to in silico* quantum chemical passage is possible; the answer is naturally positive since the previous derivation associates with the ionization process which is basically an observer intervention to the genuine quantum system, from where the final result will reflect the energetic deviation from *in se to in silico *as an irrefutable quantum manifestation of electronic system. 

Similarly, for electronic affinity, one will act on the *in se* quantum system to add an electron at the frontier level and, under the “frozen spin-orbitals” physical-chemical assumption, one gets the energetic turn from the genuine HF expression to the *in silico* orbital energy on which the “action” was undertaken (see Figure 1):

(14) EA1=EN−EN+1=−〈r|h^|r〉−∑b=1N〈rb ∣ rb〉=−εr=−εLUMO(1).

These results are usually considered as defining the popular Koopmans theorem, used for estimating the observable quantities as ionization potential and electronic affinity in terms of “artefactual” computed orbital energies (first approximation) and in the spin-orbitalic frozen framework during the electronic extraction or addition (the second approximation).

### 2.2. Compact-Finite Chemical Hardness: Koopmans' Approaches

In modern quantum chemical reactivity approaches chemical hardness plays a preeminent role due to fruitful connections it establishes with principles of hard and soft acids and bases (HSAB) and maximum hardness prescription for stabilizing a reactive compound [30–32, 36, 37]. Mutatis mutandis also gives a reliable measure for the degree of aromaticity a chemical structure displays in isolate or interaction states [63–66]. While being defined, within the conceptual density functional theory as the second-order derivative or the total *N*-electronic energy with respect to the number of changing charges, it recently acquired a significant extended working form by considering its compact-finite difference unfolding up to the third ionization and affinity order of electronic removing and attaching processes [15, 24, 36, 63]:

(15) 2η=∂2E∂N2||N〉≅2a2EN+1−2EN+EN−12+b2EN+2−2EN+EN−24 +c2EN+3−2EN+EN−39 −α2(∂2E∂N2||N−1〉  +∂2E∂N2||N+1〉) −β2(∂2E∂N2||N−2〉  +∂2E∂N2||N+2〉)=2a2EN+1−2EN+EN−12+b2EN+2−2EN+EN−24 +c2EN+3−2EN+EN−39 −α2(2a2EN−2EN−1+EN−22    +2a2EN+2−2EN+1+EN2) −β2(2a2EN−1−2EN−2+EN−32    +2a2EN+3−2EN+2+EN+12)=2a2(1+2α2−β2)  EN+1+EN−12 +(8a2β2+b2−4a2α2)EN+2+EN−24 +(c2−9a2β2)  EN+3+EN−39 −(2a2+12b2+29c2+2a2α2)EN.

Note that this expansion may in principle corresponding to the parabolic expansion of the energy containing double charged cations/anions which considerably expand the chemical reactivity analysis towards considering ceding/accepting electronic pairing (dications/dianions) or transferring entire chemical bonds in molecular interactions; while the accuracy depends on how one refines the finite differentiation schemes (see below), the limits are restricted to the frontier or valence electrons in bonding. 

The last equation may be rewritten in terms of the observational quantities, as the ionization potential (IP) and electronic affinity (EA) of the involved eigen-energies of *i*th (*i* = 1,2, 3) order

(16) $$\begin{array}{c} \text{I}{\text{P}}_{i}=E_{N-i}-E_{N-i+1}\\ \text{E}{\text{A}}_{i}=E_{N+i-1}-E_{N+i} \end{array}$$

through the energetic equivalents for the respective sums

(17) EN+1+EN−1=(IP1−EA1)+2EN,EN+2+EN−2=(IP1−EA1)+(IP2−EA2)+2EN,EN+3+EN−3  =(IP1−EA1)+(IP2−EA2)+(IP3−EA3)+2EN

to provide the working expression [15, 24, 36, 63]

(18) ηCFDIP−EA=[a2(1−α2+2β2)+14b2+19c2]IP1−EA12+[12b2+29c2+2a2(β2−α2)]IP2−EA24+[13c2−3a2β2]IP3−EA36

whose coefficients are given in Table 1, being obtained by matching the previous expansion (15) with Taylor series expansions in various orders (from second to tenth order); the results are not system dependent being susceptible to a variety of the boundary conditions [67].

It is worth remarking that when particularizing this formula for the fashioned two-point central finite difference, that is, when having *a*_2_ = 1,  *b*_2_ = *c*_2_ = *α*_2_ = *β*_2_ = 0 of Table 1, one recovers the basic chemical hardness as prescribed by the celebrated Pearson nucleophilic-electrophilic reactivity gap [20–22]

(19) $$\begin{array}{c} \eta_{2\text{C}}=\frac{\text{I}{\text{P}}_{1}-\text{E}{\text{A}}_{1}}{2} \end{array}$$

already used as measuring the aromaticity through the molecular stability against the reaction propensity [64, 65].

At this point, the third level of Koopmans' approximation may be considered, namely, through extending the second part of Koopmans' theorem as given by the identification of the IP and EA with the (minus) energies of the *in silico* highest occupied (molecular) orbital (HOMO_1_) and with the lowest unoccupied (molecular) orbital (LUMO_1_) to superior levels of HOMO_*i*=1,2,3_ and LUMO_*i*=1,2,3_, respectively,

(20) $$\begin{array}{c} \text{I}{\text{P}}_{i}=-\varepsilon_{\text{HOMO}(i)},\\ \text{E}{\text{A}}_{i}=-\varepsilon_{\text{LUMO}(i)}. \end{array}$$

With this assumption, one yields the *in silico-superior order-freezing spin-orbitals* compact-finite difference (CFD) form of chemical hardness [15, 24, 36, 63] as follows:

(21) ηCFD 
LUMO-HOMO
   =[a2(1−α2+2β2)  +14b2+19c2]   ×εLUMO(1)−εHOMO(1)2   +[12b2+29c2+2a2(β2−α2)]   ×  εLUMO(2)−εHOMO(2)4   +[13c2−3a2β2]εLUMO(3)−εHOMO(3)6.

However, one may ask whether this approximation is valid and in which conditions. This can be achieved by reconsidering the previous Koopmans first-order IP and EA to the superior differences within Hartree-Fock framework; as such, for the second order of ionization potential one gets (see Figure 1)

(22) IP2=EN−2−EN−1={∑a=1a≠ca≠dN〈a|h^|a〉+12∑a=1,b=1a≠c,b≠ca≠d,b≠dN〈ab ∣ ab〉} −{∑a=1a≠cN〈a|h^|a〉+12∑a=1,b=1a≠c,b≠cN〈ab ∣ ab〉}={∑a=1a≠ca≠dN〈a|h^|a〉+12∑a=1,b=1a≠c,b≠ca≠d,b≠dN〈ab ∣ ab〉} −{∑a=1a≠ca≠dN〈a|h^|a〉+〈d|h^|d〉+12∑a=1,b=1a≠c,b≠ca≠d,b≠dN〈ab ∣ ab〉+12∑a=1,b=1a≠c,b≠ca=d,b≠dN〈db ∣ db〉+12∑a=1,b=1a≠c,b≠ca≠d,b=dN〈ad ∣ ad〉}=−〈d|h^|d〉−∑a=1,b=1b≠dN〈db ∣ db〉=−εd=−εHOMO(2).

Note that this derivation eventually employs the equivalency for the Coulombic and exchange terms for orbitals of the same nature (with missing the same number of spin orbitals; see Figure 1). However, in the case this will be further refined to isolate the first two orders of highest occupied molecular orbitals, the last expression will be corrected with HOMO_1_/HOMO_2_ (Coulombic and exchange) interaction to successively become

(23) IP2=EN−2−EN−1=−〈d|h^|d〉 −{12∑a=1,b=1a≠c,a=d,b≠dN〈db ∣ db〉+12〈dc ∣ dc〉   +12∑a=1,b=1b≠c,a≠d,b=dN〈ad ∣ ad〉+12〈cd ∣ cd〉}=−〈d|h^|d〉−∑a=1,b=1b≠dN〈db ∣ db〉+〈cd ∣ cd〉=−εd+〈cd ∣ cd〉=−εHOMO(2)+〈HOMO1HOMO2 ∣ HOMO1HOMO2〉.

However, reloading this procedure for electronic affinity process too, one gets

(24) EA2=EN+1−EN+2=−εLUMO(2)+〈LUMO1LUMO2 ∣ LUMO1LUMO2〉.

When combining (24) with its IP counterpart (23) within the chemical hardness extended CFD analysis of (18), there appears that the simple Koopmans' orbitals energy difference is corrected by the HOMO_1_/HOMO_2_ versus LUMO_1_/LUMO_2_ as follows:

(25) IP2−EA2=εLUMO(2)−εHOMO(2) +(〈HOMO1HOMO2 ∣ HOMO1HOMO2〉−〈LUMO1LUMO2 ∣ LUMO1LUMO2〉).

This expression is usually reduced to the superior order LUMO-HOMO difference

(26) $$\begin{array}{c} \text{I}{\text{P}}_{2}-\text{E}{\text{A}}_{2}≅\varepsilon_{\text{LUMO}(2)}-\varepsilon_{\text{HOMO}(2)} \end{array}$$

due to the energetic spectra symmetry of Figure 1 relaying on the bonding versus antibonding displacements of orbitals, specific to molecular orbital theory. Therefore, with the premise that molecular orbital theory itself is correct, or at least a reliable quantum undulatory modeling of multielectronic systems moving in a nuclei potential, the above IP-EA differences in terms of Koopmans' *in silico* LUMO-HOMO energetic gaps hold also for superior orders.

An illustrative analysis for homologues organic aromatic hydrocarbons regarding how much the second and the third orders, respectively, of the IP-EA or LUMO-HOMO gaps affect the chemical hardness hierarchies, and therefore their ordering aromaticity will be exposed and discussed in the next section. 

## 3. Application on Aromatic Basic Systems

It is true that Koopmans theorem seems having some limitations for small molecules and for some inorganic complexes [44, 45]; however, one is interested here in testing Koopmans' superior orders' HOMO-LUMO behavior on the systems that work, such as the aromatic hydrocarbons. Accordingly, in Table 2 a short series of paradigmatic organics is considered, with one and two rings and various basic ring substitutions or additions, respectively [66]. For them, the HOMO and LUMO are computed, within semiempirical AM1 framework [68], till the third order of Koopmans frozen spin-orbitals' approximation; they are then combined with the various finite difference forms (from 2C to SLR) of chemical hardness as mentioned above (see Table 1) and grouped also in sequential order respecting chemical hardness gap contributions (i.e., separately for {LUMO1-HOMO1}, {LUMO1-HOMO1, LUMO2-HOMO2}, and {LUMO1-HOMO1, LUMO2-HOMO2, LUMO3-HOMO3}); the results are systematically presented in Tables 3–5. The results of Tables 3–5 reveal very interesting features, in the light of considering the aromaticity as being reliably measured by chemical hardness alone, since both associate with chemical resistance to reactivity or the terminus of a chemical reaction according to the maximum chemical hardness principle [30, 31].

Moreover, the benchmark-ordering hierarchy was chosen as produced by Hückel theory and approximation since closely related with pi-electrons delocalized at the ring level as the main source of the experimentally recorded aromaticity of organic compounds under study [69]. Note that although computational method used here is of low level, it nevertheless responds to present desiderate having a non (orbitalic) basis-dependent computational output and discussion, whereas further (Hartree-Fock) *ab initio*, (Møller-Plesset) perturbation methods, and basis set dependency considerations, as HF, MP2, and DFT, respectively, for instance, can be further considered for comparative analysis. In these conditions, the main Koopmans' analysis of chemical hardness or aromaticity behavior for the envisaged molecules leaves with relevant observations:

- In absolutely all cases, analytical or computational, the first two molecules, Benzene (I) and Pyrimidine (II), are inversed for their chemical hardness/aromaticity hierarchies respecting the benchmarking Hückel one, meaning that even in the most simple case (say 2C/{LUMO1-HOMO1}), double substitution of carbon with nitrogen increases the ring stability, most probably due to the additional pairing of electrons entering the pi-system as coming from the free valence of nitrogen atoms in molecular ring. This additional pair of electrons eventually affects by shielding also the core of the hydrocarbon rings, that is, the sigma system of Pyrimidine (II), in a specific quantum way, not clearly accounted by the Hückel theory.
- The same behavior is recorded also for the couple of molecules I and III (Pyridine), however, only for the SLR of chemical hardness computed with second and the third orders of Koopmans frozen spin-orbitals; this suggests the necessary insight the spectral like resolution analysis may provide respecting the other forms of finite compact differences in chemical hardness computation, yet only when it is combined with higher Koopmans HOMO and LUMO orbitals.
- In the same line of discussion, only for the second and the third Koopmans orders and only for the SLR chemical hardness development, that is, the last columns of Tables 4 and 5, one records similar reversed order of the molecules 2-Napthol (VII) and 1-Naphtol (VIII), with the more aromatic character for the last case when having the OH group more closely to the middy of the naphthalene structure; it is explained as previous, due to the electronic pair of chemical-bonding contribution more close to the “core” of the system with direct influence to increase the shielding electrons of the sigma systems, while leading with smoothly increased stabilization contribution (enlarging also the sigma-pi chemical gap); yet this is manifested when all the spectral-like resolution complexity is considered in chemical hardness expression and only in superior Koopmans orders (second and third), otherwise not being recorded. However, this result advocates the meaningful of considering the SLR coupled with superior Koopmans analysis in revealing subtle effects in sigma-pi aromatic systems.
- In the rest of cases the Hückel downward hierarchy of Table 2 is recovered in Tables 3–5 in a systematic way.
- When going from 2C to SLR chemical hardness analytical forms of any of Koopmans orders, on the horizontal axis through the Tables 3–5, one systematically records an increasing of the average chemical hardness/aromaticity values from 2C to 6T schemes of computations while going again down towards SLR scheme of Table 1.

**Figure 2:**
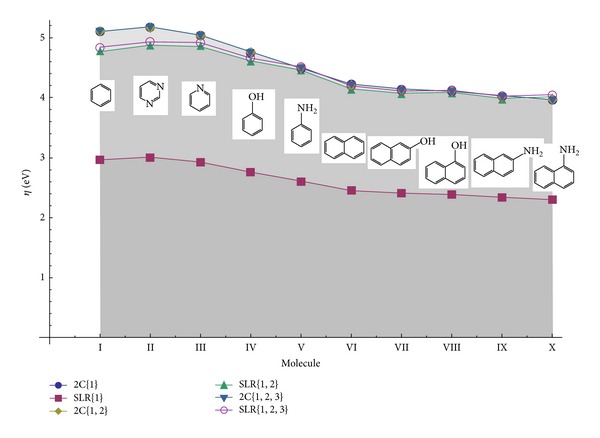
Representation of the 2C and SLR chemical hardness hierarchies for the set of molecules of Table 1 upon the first, second, and third orders of Koopmans' theorem applications as presented din Tables 3, 4, and 5, respectively.

All in all, one may compare the extreme 2C and SLR outputs of Tables 3–5 for a global view for the Koopmans' behavior respecting various orders and chemical hardness schemes of (compact-finite forms) computations: the result is graphically presented in Figure 2. The analysis of Figure 2 yields a fundamental result for the present study, that is, the practical identity among

- all Koopmans superior orbitals-based chemical hardness computations;
- the simplest 2C and the complex SLR analytical forms for compact-finite difference schemes of chemical hardness for the superior HOMO-LUMO gap extensions.

By contrary to someone expecting the first order of Koopmans theorem being more systematic, only in this order, the 2C values are practically doubled respecting SLR counterpart; such double behavior becomes convergent when superior Koopmans orders of valence orbitals are considered either in simpler or complex forms of 2C and SLR, respectively.

This may lead to the fruitful result according which the Koopmans theorem works better when superior HOMO-LUMO frozen spin orbitals are considered, probably due to compensating correlating effects that such extension implies; see analytical discussion in the last section. In any case, the present molecular illustration of Koopmans' approximations to chemical harness computation clearly shows that, at least for organic aromatic molecules, it works better for superior orders of “freezing” spin orbitals and is not limitative to the first valence orbitals, as would be the common belief. Moreover, it was also clear that the Koopmans theorem finely accords also with more complex ponder of its superior order orbitals in chemical hardness expansions equation (21), when subtle effects in lone pairing electrons (since remained orbital is frozen upon successive electronic attachment/removals on/from it) or chemical bonding pair of electrons influence the aromatic ring core towards increasing its shielding and the overall molecular reactivity resistance. All these conceptual and computational results should be further extended and tested on increased number of molecules, enlarging their variety too, as well as by considering more refined quantum computational frameworks as the density functional theory and (Hartree-Fock) ab initio schemes are currently compared and discussed for various exchange-correlation and parameterization limits and refutations. 

## 4. Conclusions

Koopmans' theorem entered on the quantum chemistry as a versatile tool for estimating the ionization potentials for closed-shells systems, and it was widely confirmed for organic molecular systems, due to the inner usually separation between sigma (core) and pi (valence) subelectronic systems, allowing to treat the “frozen spin orbitals” as orbitals not essentially depending on the number of electrons in the valence shells, when some of them are extracted (via ionization) or added (via negative attachments); this approximation ultimately works for Hartree-Fock systems when electronic correlation may be negligible or cancels with the orbital relaxations during ionization or affinity processes, respectively; naturally, it works less when correlation is explicitly counted, as in density functional theory, where instead the exchange energies are approximated or merely parameterized so that “loosing” somehow on the genuine spin-orbital nature of the mono-determinantal approach of the Hartree-Fock, with a natural energetic hierarchy included. Despite the debating context in which Koopmans theorem is valid or associates with a physical-chemical sense, the present work gives some insight in this matter by clarifying upon some key features of Koopmans analysis, namely;

- the Hartree-Fock spin orbitals involved in Koopmans' theorem are of computational nature, emerged through solving an eigen-problem in a given basis set, so that being characterized by a sort of “quantum shift” related with quantum uncertainty when the free system is affected by observation—here by computation, so this behavior is at its turn computationally naturally and not viewed as a conceptual error in structurally assessing a many-electronic structure;
- the Koopmans theorem not restrictedly refers to the first ionization potential and may be extended to successive ionization potentials (and electronic affinities) as far the valence shell is not exhausted by the pi-collective electrons, such that the sigma-pi separation may be kept reliable and the “frozen spin-orbitals” may be considered as such through cancellation of the relaxation effects with the electronic correlations, both explicitly escaping to Hartree-Fock formalism; this was, however, here emphasized by the appearance of the quantum terms of type 〈HOMO_1_HOMO_2_ | HOMO_1_HOMO_2_〉 in (23) and 〈LUMO_1_LUMO_2_ | LUMO_1_LUMO_2_〉 in (24) which were considered as reciprocal annihilating in chemical hardness' IP-EA differences in (25) due to symmetrical bonding versus antibonding spectra displacements in molecular orbital theory—as a simplified version of Hartree-Fock theory;
- the Koopmans theorem goes at best with chemical harness or aromaticity evaluation by means of LUMO-HOMO gaps when they manifested surprisingly the same for superior orders of IPs-EAs, this way confirming the previous point.

Application on a paradigmatic set of mono and double benzoic rings molecules supported these conclusions, yet leaving enough space for further molecular set extensions and computational various frameworks comparison.

## Figures and Tables

**Table 1:** Numerical parameters for the compact finite second (2C)-, fourth (4C)-, and sixth (6C)-order central differences; standard Padé (SP) schemes; sixth (6T)- and eight (8T)-order tridiagonal schemes; eighth (8P)- and tenth (10P)-order pentadiagonal schemes up to spectral-like resolution (SLR) schemes for chemical hardness of (18) [15, 24, 36, 63].

Scheme	*a*_2_	*b*_2_	*c*_2_	*α*_2_	*β*_2_
2C	1	0	0	0	0
4C	1.333	−0.333	0	0	0
6C	1.091	0.273	0	0.182	0
SP	1.2	0	0	0.1	0
6T	1.5	−0.6	0.2	0	0
8T	0.967	0.537	−0.03	0.237	0
8P	0.814	0.789	0	0.292	0.01
10P	0.592	1.155	0.044	0.372	0.024
SLR	0.216	1.723	0.177	0.502	0.056

**Table 2:** Molecular structures of paradigmatic aromatic hydrocarbons [66], ordered downwards according with their Hückel first-order HOMO-LUMO gap [69], along their first three highest occupied (HOMOs) and lowest unoccupied (LUMOs) (in electron-volts (eV)) computationally recorded levels within semiempirical AM1 method [68].

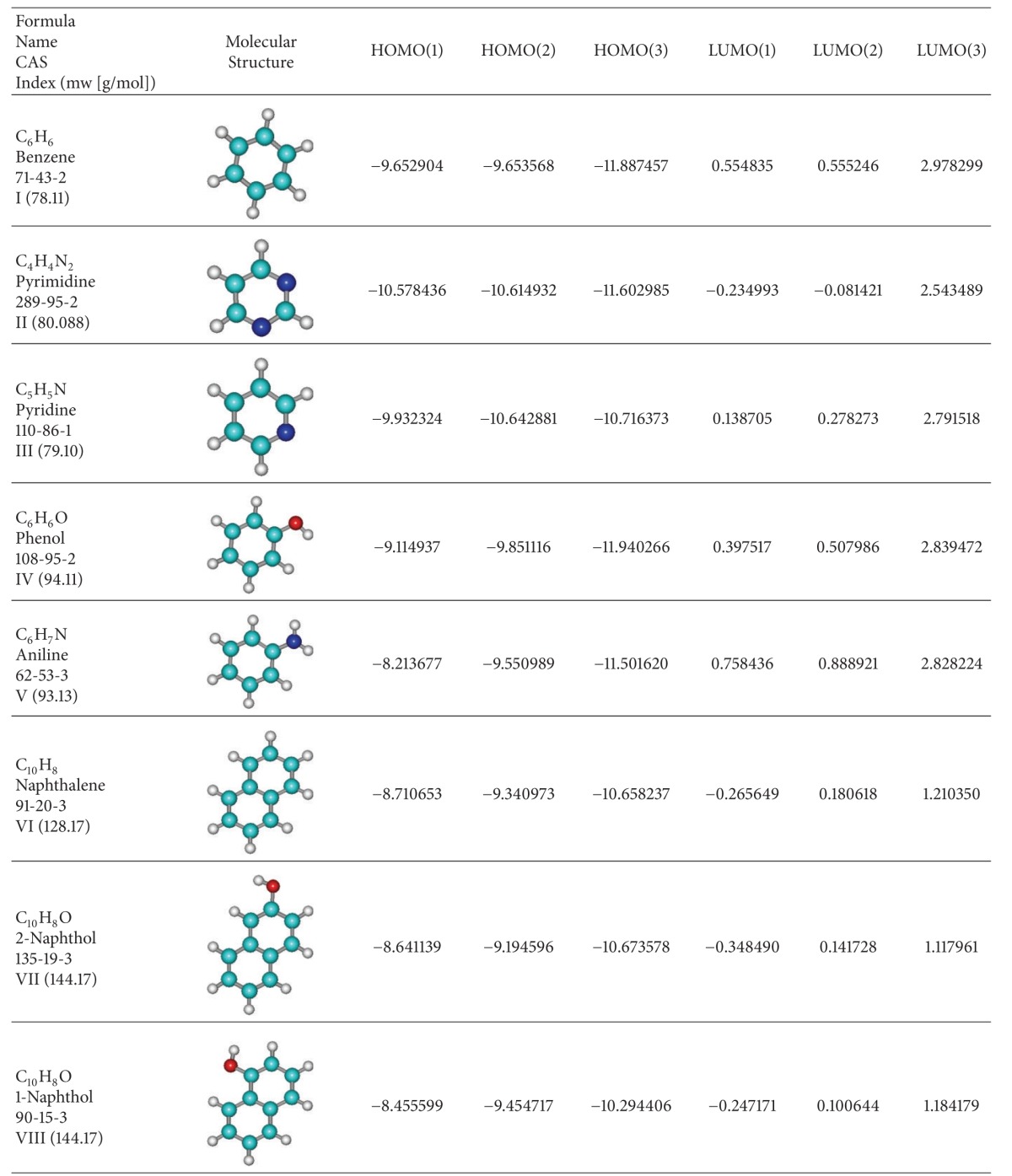 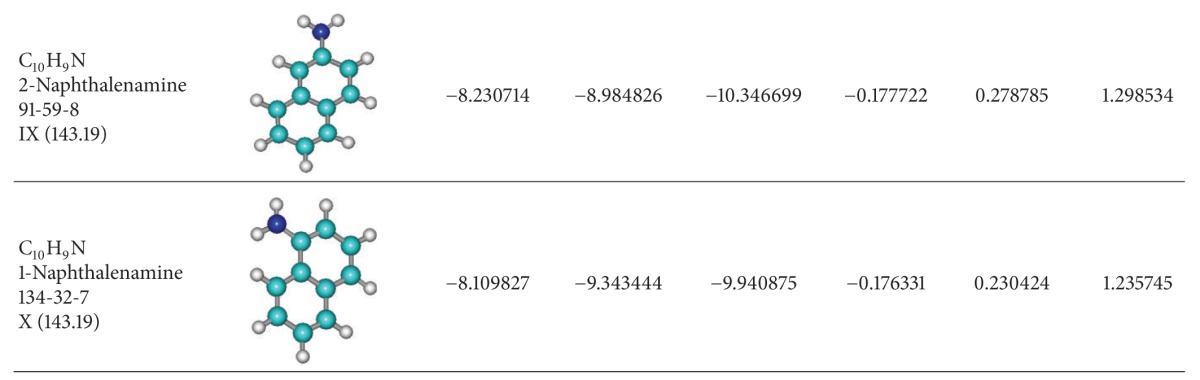

**Table 3:** Chemical hardness values (in eV) as computed for molecules of Table 2 with first-order LUMO(1)-HOMO(1) gap order of (21) with parameters of Table 1.

Molecule	*η*_2C_	*η*_4C_	*η*_6C_	*η*_SP_	*η*_6T_	*η*_8T_	*η*_8P_	*η*_10P_	*η*_SLR_
I	5.10387	6.379837	4.903511	5.512179	7.003643	4.434762	4.030827	3.542746	2.971354
II	5.171722	6.464652	4.968699	5.585459	7.096751	4.493719	4.084414	3.589844	3.010856
III	5.035515	6.294393	4.837839	5.438356	6.909845	4.375368	3.976843	3.495299	2.931559
IV	4.756227	5.945284	4.569516	5.136725	6.5266	4.132695	3.756273	3.301437	2.768964
V	4.486057	5.607571	4.309951	4.844941	6.155866	3.897943	3.542904	3.113904	2.611677
VI	4.222502	5.278128	4.056743	4.560302	5.794211	3.66894	3.334759	2.930963	2.458242
VII	4.146325	5.182906	3.983556	4.47803	5.689679	3.60275	3.274597	2.878086	2.413893
VIII	4.104214	5.130268	3.943098	4.432551	5.631894	3.56616	3.24134	2.848856	2.389378
IX	4.026496	5.03312	3.868431	4.348616	5.525247	3.49863	3.179962	2.794909	2.344132
X	3.966748	4.958435	3.811029	4.284088	5.44326	3.446715	3.132775	2.753437	2.309348

**Table 4:** Chemical hardness values (in eV) as computed for molecules of Table 2 with first-order LUMO(1)-HOMO(1) and second-order LUMO(2)-HOMO(2) gaps of (21) with parameters of Table 1.

Molecule	*η*_2C_	*η*_4C_	*η*_6C_	*η*_SP_	*η*_6T_	*η*_8T_	*η*_8P_	*η*_10P_	*η*_SLR_
I	5.10387	5.95447	4.239094	4.89965	6.351413	3.933493	3.865279	3.990091	4.778726
II	5.171722	6.025756	4.283151	4.953449	6.423777	3.976506	3.9136	4.051417	4.875712
III	5.035515	5.839345	4.127062	4.783086	6.212105	3.839122	3.799743	3.973858	4.865044
IV	4.756227	5.513655	3.895318	4.515179	5.864769	3.624046	3.588288	3.755367	4.602943
V	4.486057	5.172574	3.630494	4.218546	5.488872	3.385327	3.373608	3.571375	4.459963
VI	4.222502	4.881395	3.437052	3.989007	5.185887	3.201415	3.180355	3.348194	4.143948
VII	4.146325	4.793892	3.375923	3.917851	5.093191	3.144321	3.123197	3.287199	4.066799
VIII	4.104214	4.732127	3.32121	3.859229	5.021412	3.096976	3.086388	3.267567	4.081062
IX	4.026496	4.647136	3.265531	3.792799	4.933405	3.043772	3.029741	3.200836	3.984165
X	3.966748	4.559524	3.187936	3.709656	4.831596	2.976622	2.977523	3.172958	4.004309

**Table 5:** Chemical hardness values (in eV) as computed for molecules of Table 2 with first-order LUMO(1)-HOMO(1), second-order LUMO(2)-HOMO(2), and third-order LUMO(3)-HOMO(3) gaps of (21) with parameters of Table 1.

Molecule	*η*_2C_	*η*_4C_	*η*_6C_	*η*_SP_	*η*_6T_	*η*_8T_	*η*_8P_	*η*_10P_	*η*_SLR_
I	5.10387	5.95447	4.239094	4.89965	6.516588	3.908499	3.806245	3.921086	4.834997
II	5.171722	6.025756	4.283151	4.953449	6.58096	3.952722	3.857423	3.985751	4.929261
III	5.035515	5.839345	4.127062	4.783086	6.362192	3.816411	3.746102	3.911156	4.916176
IV	4.756227	5.513655	3.895318	4.515179	6.028988	3.599197	3.529596	3.686762	4.658889
V	4.486057	5.172574	3.630494	4.218546	5.648093	3.361234	3.316702	3.504858	4.514206
VI	4.222502	4.881395	3.437052	3.989007	5.31776	3.18146	3.133223	3.293101	4.188874
VII	4.146325	4.793892	3.375923	3.917851	5.224208	3.124496	3.076372	3.232464	4.111434
VIII	4.104214	4.732127	3.32121	3.859229	5.148952	3.077677	3.040805	3.214284	4.124512
IX	4.026496	4.647136	3.265531	3.792799	5.062797	3.024193	2.983496	3.14678	4.028246
X	3.966748	4.559524	3.187936	3.709656	4.955781	2.957831	2.933139	3.121078	4.046616

## Abbreviations

*η*: Chemical hardness
CFD: Compact-finite difference
EA: Electronic affinity
HOMO: Highest occupied molecular orbital
HSAB: Hard-and-soft acids and bases
IP: Ionization potential
KT: Koopmans theorem
LUMO: Lowest unoccupied molecular orbital
SLR: Spectral like resolution
DFT: Density functional theory.
